# Lipid levels and risk of acute pancreatitis using bidirectional Mendelian randomization

**DOI:** 10.1038/s41598-024-56946-x

**Published:** 2024-03-15

**Authors:** Biqi Wang, Jacqueline S. Dron, Yuxuan Wang, Seung Hoan Choi, Jennifer E. Huffman, Kelly Cho, Peter W. F. Wilson, Pradeep Natarajan, Gina M. Peloso

**Affiliations:** 1 Department of Biostatistics, Boston University School of Public Health, Boston, MA USA; 2 Department of Medicine, University of Massachusetts Chan Medical School, Worcester, MA USA; 3 Center for Genomic Medicine, Massachusetts General Hospital, Boston, MA USA; 4 Program in Medical and Population Genetics, Broad Institute of Harvard and MIT, Cambridge, MA USA; 5 Veterans Affairs Healthcare System, Boston, MA USA; 6 Division of Aging, Department of Medicine, Mass General Brigham, Harvard Medical School, Boston, MA USA; 7 Atlanta Veterans Affairs Medical Center, Decatur, GA USA; 8 Division of Cardiology, Department of Medicine, Emory University School of Medicine, Atlanta, GA USA; 9 Department of Epidemiology, Rollins School of Public Health, Emory University, Atlanta, GA USA; 10 Cardiovascular Research Center, Massachusetts General Hospital, Boston, MA USA; 11 Department of Medicine, Harvard Medical School, Boston, MA USA

**Keywords:** Lipids, Acute pancreatitis, Mendelian randomization, Genetic association study, Acute pancreatitis

## Abstract

Previous studies found lipid levels, especially triglycerides (TG), are associated with acute pancreatitis, but their causalities and bi-directions were not fully examined. We determined whether abnormal levels of TG, high-density lipoprotein cholesterol (HDL-C), and low-density lipoprotein cholesterol (LDL-C) are precursors and/or consequences of acute pancreatitis using bidirectional two-sample Mendelian randomization (MR) with two non-overlapping genome-wide association study (GWAS) summary statistics for lipid levels and acute pancreatitis. We found phenotypic associations that both higher TG levels and lower HDL-C levels contributed to increased risk of acute pancreatitis. Our GWAS meta-analysis of acute pancreatitis identified seven independent signals. Genetically predicted TG was positively associated with acute pancreatitis when using the variants specifically associated with TG using univariable MR [Odds ratio (OR), 95% CI 2.02, 1.22–3.31], but the reversed direction from acute pancreatitis to TG was not observed (mean difference = 0.003, SE = 0.002, *P*-value = 0.138). However, a bidirectional relationship of HDL-C and acute pancreatitis was observed: A 1-SD increment of genetically predicted HDL-C was associated with lower risk of acute pancreatitis (OR, 95% CI 0.84, 0.76–0.92) and genetically predisposed individuals with acute pancreatitis have, on average, 0.005 SD lower HDL-C (mean difference = − 0.005, SE = 0.002, *P*-value = 0.004). Our MR analysis confirms the evidence of TG as a risk factor of acute pancreatitis but not a consequence. A potential bidirectional relationship of HDL-C and acute pancreatitis occurs and raises the prospect of HDL-C modulation in the acute pancreatitis prevention and treatment.

## Background

Abnormal blood lipid levels are well-established risk factors for cardiovascular disease and all-cause mortality^1,2^, but the association of lipid concentrations with diseases of the pancreas, especially acute pancreatitis, is controversial^3,4^. Increased triglyceride (TG) levels have been associated with augmented risk of acute pancreatitis in observational studies, and hypertriglyceridemic pancreatitis is a common cause of acute pancreatitis in people with high TG levels [≥ 500 mg/dL (or ≥ 5.65 mmol/L)]^4^. Previous studies have suggested genetically predicted TG levels would increase the risk of acute pancreatitis^5–7^. Conversely, acute pancreatitis can injure the pancreas, adversely affect lipid metabolism and elevate TG levels^8,9^. The question remains whether acute pancreatitis is a risk factor for high TG or high TG is a risk factor for acute pancreatitis. Additionally, there is limited evidence on the associations between high-density lipoprotein cholesterol (HDL-C), low-density lipoprotein cholesterol (LDL-C), and acute pancreatitis: decreased HDL-C associated with high risk of severe acute pancreatitis and a U-shape association for LDL-C levels and severe acute pancreatitis^4,10^.

Mendelian Randomization (MR) is a statistical genetic technique to infer causal relationships between traits^11,12^. Bidirectional Mendelian randomization (bi-MR) can limit confounding and be used to determine the direction(s) of effect between two traits with the use of appropriate genetic variants^13,14^. In this study we perform bi-MR to determine whether lipid levels (TG, HDL-C, LDL-C) are precursors or consequences of acute pancreatitis.

## Results

### Association of lipids with acute pancreatitis

**Figure 1:**
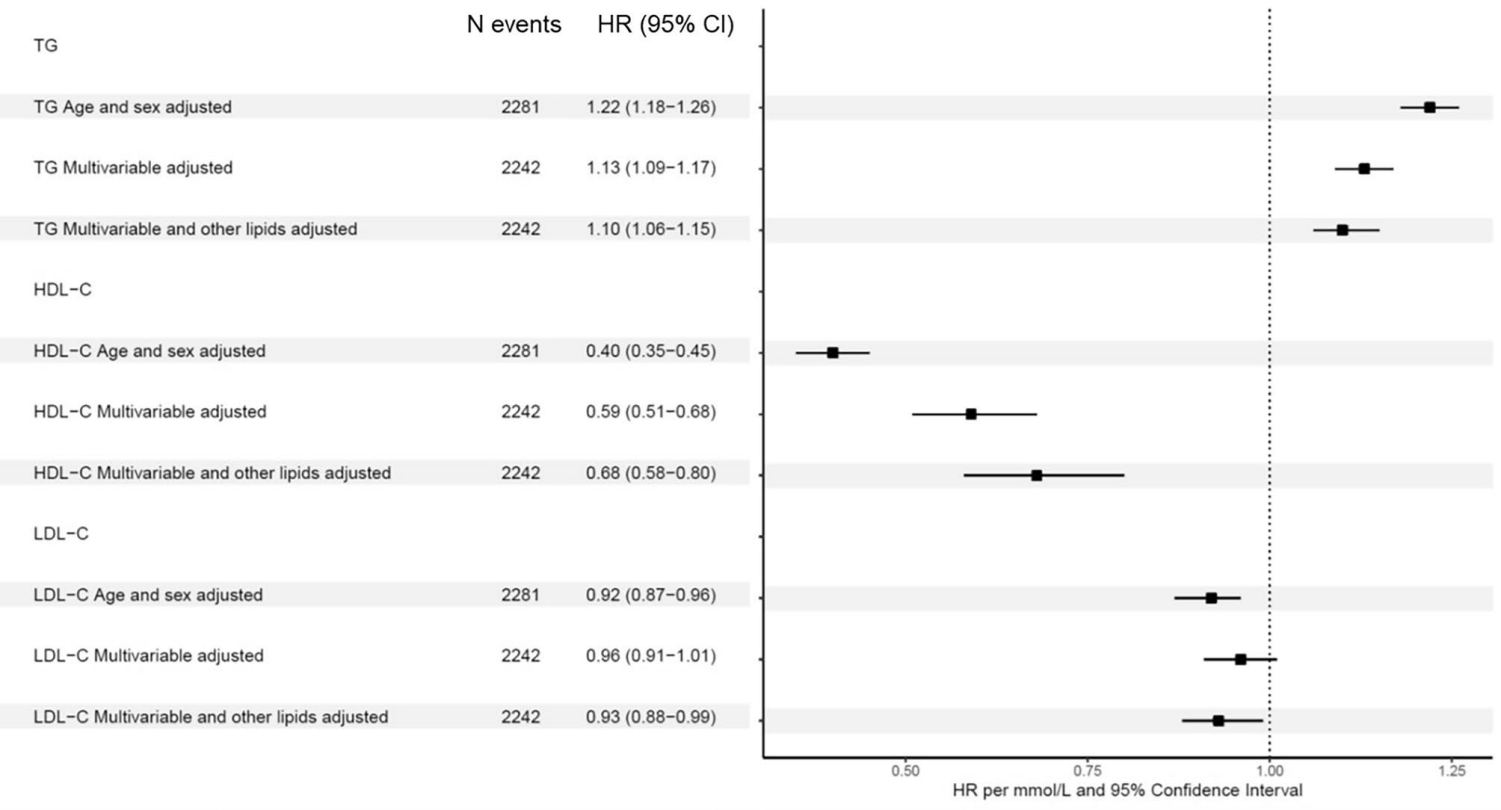
Phenotypic association of lipids and incident acute pancreatitis in the UK Biobank. Covariates in the multivariable adjusted model were age, sex, baseline BMI, calcium level, smoking status, drinking, pancreatitis at risk medication, lipid-lowering medication, diabetes (type 1 and 2), and prevalent cardiovascular diseases.

We identified 2281 incident acute pancreatitis cases among the 421,181 eligible participants from the UK Biobank. The acute pancreatitis incident cases had a worse lipid profile, less ideal smoking and drinking behaviors, and more comorbidities than the controls (Table 1). TG was positively associated with incident acute pancreatitis in the multivariable and other lipid adjusted model [hazard ratio (HR, 95% CI) 1.10, (1.06–1.15)], while per 1 mmol/L HDL-C increment is associated with lower acute pancreatitis risk by 32% in the fully adjustment model [HR, 95% CI 0.68, (0.58–0.80)]. Although we found a marginal negative association between LDL-C and acute pancreatitis risk, such associations were not robust in the multivariable adjusted model (Fig. 1).

**Table 1:** Baseline characteristics of individuals for lipids and incident acute pancreatitis in the UK Biobank.

	Overall	Controls	Incident cases
n	421,181	418,900	2,281
Age, years (mean (SD))	56.51 (8.10)	56.50 (8.10)	58.55 (7.78)
Males (%)	194,773 (46.2)	193,641 (46.2)	1132 (49.6)
Triglycerides, mmol/L (mean (SD))	1.74 (1.02)	1.74 (1.02)	2.03 (1.17)
HDL-C, mmol/L (mean (SD))	1.45 (0.38)	1.45 (0.38)	1.34 (0.37)
LDL-C, mmol/L (mean (SD))	3.56 (0.87)	3.56 (0.87)	3.50 (0.91)
BMI, kg/m^2^ (mean (SD))	27.42 (4.78)	27.41 (4.77)	29.50 (5.47)
Smoking (%)			
Never	229,539 (54.8)	228,447 (54.8)	1092 (48.2)
Previous	145,129 (34.6)	144,272 (34.6)	857 (37.9)
Current	44,368 (10.6)	44,053 (10.6)	315 (13.9)
Alcohol, drinks/day (mean (SD))*	3.81 (4.56)	3.81 (4.56)	3.61 (5.34)
Calcium, mmol/L (mean (SD))	2.38 (0.09)	2.38 (0.09)	2.38 (0.10)
AP induced medication use (%) †	1428 (0.3)	1406 (0.3)	22 (1.0)
With lipid-lowering medication (%)	69,838 (16.6)	69,279 (16.5)	559 (24.5)
Prevalent diabetes (type 1 and 2) (%)	9215 (2.2)	9127 (2.2)	88 (3.9)
Prevalent CVD (%)	19,717 (4.7)	19,508 (4.7)	209 (9.2)
Incident acute pancreatitis (%)	2281 (0.5)	0 (0.0)	2281 (100.0)
Follow-up years (mean (SD))	12.52 (1.74)	12.56 (1.67)	6.88 (3.64)

*One drink equals to 14 g or 0.6 oz pure alcohol.

†AP induced medications includes the medications (e.g., some immunosuppressive agents) that might induce acute pancreatitis such as azathioprine or 6-mercaptopurine.

AP: acute pancreatitis.

### Acute pancreatitis GWAS

**Figure 2:**
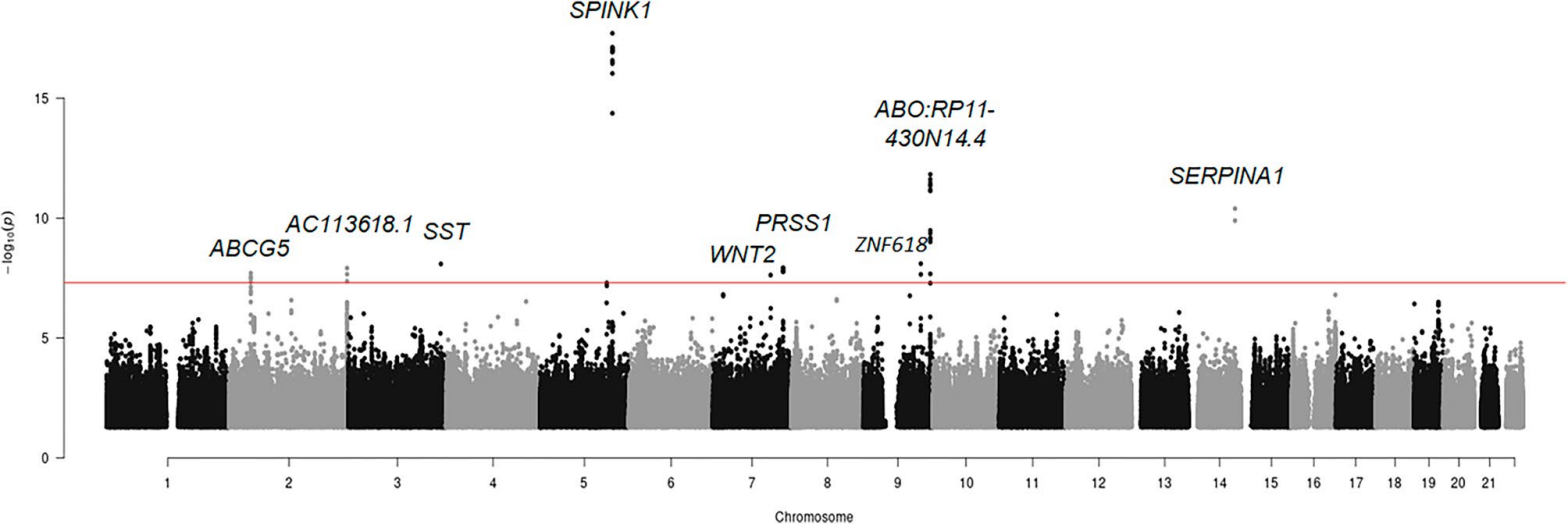
Manhattan plot of acute pancreatitis in the GWAS meta-analysis of the UK Biobank and the MVP.

The descriptive statistics of acute pancreatitis and controls of UK Biobank and Million Veteran Program (MVP) genome-wide association studies (GWAS) are shown in Supplemental Tables 1 and 2. The GWAS from UK Biobank alone identified 3 variants associated with acute pancreatitis and the top signal was a variant in the intron of the *ABCG5* (Supplemental Fig. 1 Panel A). When we meta-analyzed MVP GWAS and UK Biobank, we observed nine independent acute pancreatitis associated variants, with the top signal near *SPINK1* (Table 2, Fig. 2, and Supplemental Table 3). A test of heterogeneity suggested heterogeneity occurred only in the variant of *ABCG5* (Supplemental Table 3). We did not observe inflation in the UK Biobank GWAS nor the meta-analysis based on the quantile–quantile plots (Supplemental Fig. 1 Panel B, and Panel C).

**Table 2:** Genome-wide significant variants (*P*-value < 5e−08) in the meta-analysis results of acute pancreatitis GWAS in UK Biobank and MVP.

Variants	Effect allele	Other allele	EAF	N	Effect size	S.E	*P*-value	Direction	Nearest Gene	Distance to nearest gene (bp)	Function	CADD
rs10901252	C	G	0.09	1,112,238	0.14	0.02	1.52E−12	+ +	*ABO:RP11-430N14.4*	0:0	ncRNA exonic	0.20
rs113812546	A	C	0.02	1,112,238	0.22	0.04	2.39E−08	+ +	*WNT2*	4494	Intergenic	0.41
rs117046683	A	C	0.55	1,112,238	0.07	0.01	1.20E−08	+ +	*PRSS1*	2664	Intergenic	0.40
rs142703147	A	C	0.01	1,112,238	0.48	0.05	2.02E−18	+ +	*SPINK1*	3931	Intergenic	14.58
rs28929474	T	C	0.02	1,112,238	0.29	0.04	4.04E−11	+ +	*SERPINA1*	0	Exonic	20.20
rs35471107	C	G	0.56	1,112,238	0.08	0.01	8.22E−09	+ +	*SST*	37,465	Intergenic	0.64
rs74355737	A	C	0.99	170,754	− 0.59	0.10	2.25E−08	?-	*ZNF618*	0	Intronic	0.83
rs77725792	A	G	0.07	1,112,238	0.13	0.02	2.00E−08	+ +	*ABCG5*	0	Intronic	0.49
rs9677610	A	G	0.45	1,112,238	− 0.07	0.01	1.23E−08	–	*AC113618.1*	7433	Intergenic	1.89

*EAF* Effect allele frequency, *S.E* Standard Error, *bp* base pairs, *ncRNA* non-coding RNA, *CADD* Combined annotation dependent depletion.

### Univariable MR from each lipid levels to acute pancreatitis

**Figure 3:**
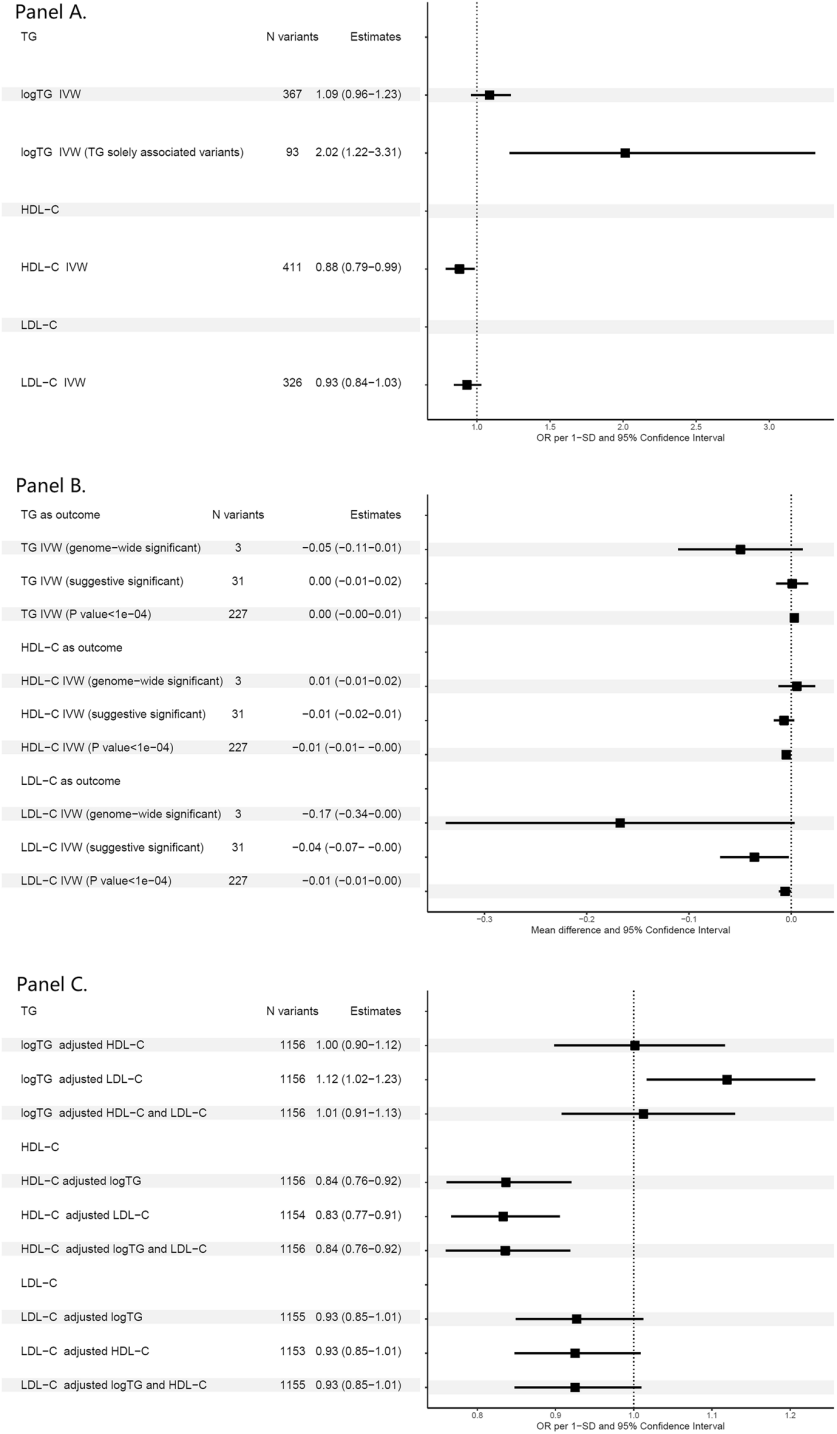
Bidirectional MR results of lipids and acute pancreatitis. Panel (**A**). Univariable MR from each lipids to acute pancreatitis; Panel (**B)**. Univariable MR from acute pancreatitis to each lipid outcomes with varied IV sets; Panel (**C)**. Multivariable MR from lipids to acute pancreatitis.

The associations of genetically predicted HDL-C or genetically predicted LDL-C with acute pancreatitis from the univariable MR were similar to the results of multivariable MR: negative associations between HDL-C and acute pancreatitis, and a null association of LDL-C and acute pancreatitis (Fig. 3 Panel A). Horizontal pleiotropy was not identified in the instrumental variables for HDL-C (MR Egger intercept = − 0.002, SE = 0.002, *P*-value = 0.232) or LDL-C (MR Egger intercept = − 0.001, SE = 0.002, *P*-value = 0.574). However, strong horizontal pleiotropy was observed with the variants as instruments for TG (MR Egger intercept = 0.008, SE = 0.002, *P*-value = 0.0001). The genetically predicted TG was not associated with acute pancreatitis in the univariable IVW model when pleiotropic variants were included. We then used the 66 variants that were solely associated with TG as the instruments and the genetically predicted 1-SD increment in natural log-transformed TG was associated with a 2.02-fold increase in odds of having acute pancreatitis (OR 95% CI 1.22–3.31).

The overall Steiger directional test from the HDL-C to acute pancreatitis indicated the direction was true by those 411 variants (R^2^ of HDL-C: 4.16%, R^2^ of acute pancreatitis: 0.11%, *P*-value < 2.23e−308). And, the direction from TG to acute pancreatitis by the 93 solely-associated variants was also observed (R^2^ of logTG: 0.45%, R^2^ of acute pancreatitis: 0.03%, *P*-value = 4.53e−182).

Sensitivity analysis using other MR methods such as MR-Egger, simple mode MR, weighted median, weighted mode MR, and MR-PRESSO, were mostly consistent with the IVW results (Supplemental Table 4). Heterogeneity among variants as the instrumental variables was observed in all lipids (Supplemental Table 4). After filtering out the variants by variants’ Steiger test (i.e., direction should be from lipid to acute pancreatitis and *P*-value of Steiger test for a specific variant should less than 0.05), we did not observe heterogeneity among the remaining variants, however, the averaged effect sizes were slightly attenuated comparing to the nonfiltered results.

For the fifteen *LPL* pathway variants previously used as the instrumental variable of TG to acute pancreatitis, only 12 of them were available in the GLGC summary statistics without the UK Biobank. Five of the 12 variants were rare, with allele frequencies less than 1%. Although the 12 variants were strongly associated with TG, their predicted TG was not associated with acute pancreatitis in any univariable MR methods (Supplemental Table 5).

### Reversed univariable MR from acute pancreatitis to each lipid

The results of reversed univariable MR depend on the choices of acute pancreatitis-associated variants that were used as instrumental variables. When we used either 3 genome-wide significant variants, or 31 suggestive significant variants as the instruments (*P*-value < 1e−05), the genetically predisposed acute pancreatitis was not associated with any lipid outcomes (Fig. 3 Panel B). However, when we included additional suggestive pancreatitis associated variants (i.e., *P*-value < 1e−04 in the UK Biobank, with some replication in the MVP GWAS), the genetically predisposed acute pancreatitis averaged 0.005 SD lower HDL-C (mean difference = − 0.005, SE = 0.002, *P*-value = 0.004). Such associations were robust when we used varied MR methods, considered the Steiger’s filtering, and removed the *ABCG5* variant which might be heterogeneous in the meta-analysis (Supplemental Table 6). The overall Steiger test from the acute pancreatitis to the HDL-C by the 227 variants also suggested the reversed causation existed (R^2^ of acute pancreatitis: 0.80%, R^2^ of HDL-C: 0.20%, *P*-value = 2.13e−143).

### Multivariable MR from lipid levels to acute pancreatitis

We observed consistent results that 1-SD increment of genetically predicted HDL-C lowered the risk of acute pancreatitis after TG and/or LDL-C adjustment in the multivariable MR models (Fig. 3 Panel C, Odds ratio (OR), 95% CI 0.84, 0.76–0.92). Although the genetically predicted TG was positively associated with acute pancreatitis after LDL-C adjustment, such association attenuated to null when HDL-C was added to the model. We did not observe any associations between genetically predicted LDL-C and acute pancreatitis in the multivariable models (Fig. 3 Panel C).

## Discussion

We observed positive phenotypic associations between TG and acute pancreatitis and negative associations for HDL-C in over 420,000 UK Biobank individuals, as has previously been reported^15,16^. Using MR, our findings suggested that both higher TG levels and lower HDL-C levels contribute to increased risk of acute pancreatitis. The reversed-MR analysis provided no consistent evidence of pancreatitis as a risk factor for abnormal lipid levels, but acute pancreatitis status might result in an HDL-C decrement.

Our univariable MR analysis of TG to acute pancreatitis was consistent with previous MR findings and we additionally observed HDL-C and acute pancreatitis associations that previous MR did not^6,7^. The inconsistent HDL-C results might be based on the choice of GWAS summary statistics and the genetic variants selected as instrumental variables. Comparing with previous analysis, we used the summary statistics with larger sample sizes and more HDL-C associated variants, thus our analysis was more powerful to observe significant associations^17^.

The results from our MR analysis emphasizes that clinical strategies to control lipids levels should favorably affect acute pancreatitis risk. Previous meta-analysis of randomized clinical trials showed that the use of statin therapy was associated with a lower risk of pancreatitis in patients with normal or mildly elevated TG levels^18^. Although those trials did not separate acute or chronic pancreatitis endpoints, we speculate their findings were mostly driven by acute pancreatitis, as acute onset is more common^15^. Experimental models support the lipids pathogenesis of acute pancreatitis in the inflammatory processes^19–21^. In the reversed MR, we found acute pancreatitis might causally lead to lower level of HDL-C. Mechanistically, several inflammatory stimulators might reduce the expression of Apo A1, leading to a drop in HDL-C or reverse cholesterol transport process might be impaired, resulting in decreased cholesterol efflux and an increase in intracellular cholesterol content^22–25^. Our bidirectional MR offers additional evidence that lipids may be a risk factor in acute pancreatitis, and acute pancreatitis may worsen the lipid levels through initiating inflammatory processes.

We also observed from our multivariable and univariable MR that the associations of genetically predicted TG and acute pancreatitis may have been masked by the presence of HDL-C. TG and HDL-C are highly negatively correlated (Pearson correlations range from − 0.26 to − 0.58)^26^ and over 65% (241/367) TG associated variants are also associated with HDL-C (i.e., *P*-value for TG < 5e−08 and *P*-value for HDL-C < 1e−04). The MR Egger intercept test showed that TG selected variants exhibited horizontal pleiotropy, so minimizing the pleiotropy caused by HDL-C in the TG and acute pancreatitis association was crucial. Similar findings of TG and coronary heart diseases associations that attenuated or even disappeared when adjusted for HDL-C were also reported by previous epidemiology studies^26,27^, implicating the weak associations of TG and heart diseases were more likely to be outweighed by measurement considerations such as larger biological variations dependent on other factors such as diet, prior alcohol intake, and blood sample collections^28^. The effect size of HDL-C diminished with TG adjustments, which partially supports the evidence of TG as one risk factor for acute pancreatitis.

We identified nine acute pancreatitis variants in the meta-analysis of UK Biobank and the MVP. We successfully replicated the variant in the non-coding regions at the *PRSS1-PRSS2* gene, which was previously reported by an acute pancreatitis GWAS^29^. We also confirmed the findings of variants around *ABCG5* gene associated with acute pancreatitis by a recent GWAS meta-analysis which including the UK Biobank^30^. The other variants we identified were associated with pancreatitis and liver disease. For example, we found exotic variants in *SERPINA1* gene which encode the protein associated with chronic obstructive pulmonary disease, emphysema, and chronic liver disease^31,32^. And we also found intergenic variants near *SPINK1* gene whose mutations are associated with hereditary pancreatitis and tropical calcific pancreatitis^30^.

The strengths of our study include using a two-sample non-overlapping MR design to avoid the over-fitting issue that can occur in one-sample MR. Weak instrument bias in the one-sample MR will be expected to bias towards a confounded result^33^. The summary statistics provided from large consortiums reassure the strengths of the instrumental variables. Secondly, our MR analysis was confirmed by multiple sensitivity analysis to evaluate the effects of pleiotropy, and to provide more reliable and robust results across varied methods. Thirdly, beyond the previous study focusing on TG and its causality to pancreatitis disease, we included other two lipids (HDL-C, and LDL-C) into the MR analysis and mutually adjusted them and examined the reversed causation from acute pancreatitis to lipids.

We acknowledge several limitations. Firstly, we assumed the associations of lipids and acute pancreatitis were linear, however there might be non-linear associations^34^, especially for LDL-C. Secondly, the number of acute pancreatitis cases was limited, and our analyses may lack power to detect more acute pancreatitis associated variants. Thirdly, although the summary statistics were based on multi-ancestral GWAS, the majority population of the GWAS was Europeans, the generalizability of our study should be confirmed in other populations. Lastly, the form of confounding specific to MR, usually referred to as “horizontal” pleiotropy, could have biased our findings if some of the variants used as proxies for lipids were also associated with other traits (e.g., obesity, diabetes, alcohol consumption, smoking, and diet etc.) affecting acute pancreatitis^6,7,35^. Although we have tried to control such pleiotropy by several sensitivity analysis and using the solely associated variants, we could not guarantee those instrumental variants were not associated with other unknown factors. Indeed, one of the top causes of acute pancreatitis is "idiopathic", with multiple other less common etiologies to follow given the complex, multi-etiological, and highly heterogeneous nature of acute pancreatitis^36,37^.

## Conclusions

In conclusion, higher levels of TG or lower levels of HDL-C are risk factors for acute pancreatitis. On the contrary, there was no consistent evidence that acute pancreatitis causally modifies lipid levels. Lipid management (e.g., lowering-lipid drug usage) may prevent acute pancreatitis or could be a part of acute pancreatitis patient treatment.

## Methods

### Study populations

We examined the phenotypic associations between baseline lipids (TG, HDL-C, and LDL-C) and risk of acute pancreatitis in 421,181 UK Biobank participants (Supplemental Methods, Supplemental Fig. 2, Supplemental Table 7)^15,38,39^. We then conducted a two-sample MR analysis in two non-overlapping populations. For lipid levels, we used the Global Lipids Genetics Consortium (GLGC) GWAS results without the UK Biobank data comprising over one million individuals from over 200 cohorts across diverse ancestries^40^. For pancreatitis data, we used data from the UK Biobank (Supplemental Methods)^41^. To boost the power to identify acute pancreatitis associated variants, we also performed a meta-analysis for acute pancreatitis with the UK Biobank and the MVP GWAS (Supplemental Methods)^42^.

The overall study was approved by the institutional review board (IRB) of the Boston University Medical Center. Individual studies were approved by the appropriate IRBs and informed consent was obtained from all participants. Secondary use of the UK Biobank data was facilitated through UKB application 42,614. This study has been performed in accordance with the Declaration of Helsinki and all research was performed in accordance with relevant guidelines.

### Association of lipid levels and risk of acute pancreatitis

For phenotypic associations of each baseline lipid and incident pancreatitis, we performed age and sex adjusted Cox proportional hazards models in the UK Biobank. The associations were additionally adjusted for baseline BMI, calcium level, smoking status, drinking, pancreatitis at risk medication which might induce acute pancreatitis such as azathioprine or 6-mercaptopurine, lipid-lowering medication, diabetes (type 1 and 2), and prevalent cardiovascular diseases. We also ran a third multivariable model in which we adjusted for all lipid measures in addition to all previous covariates.

### Selection of instrumental variables

**Figure 4:**
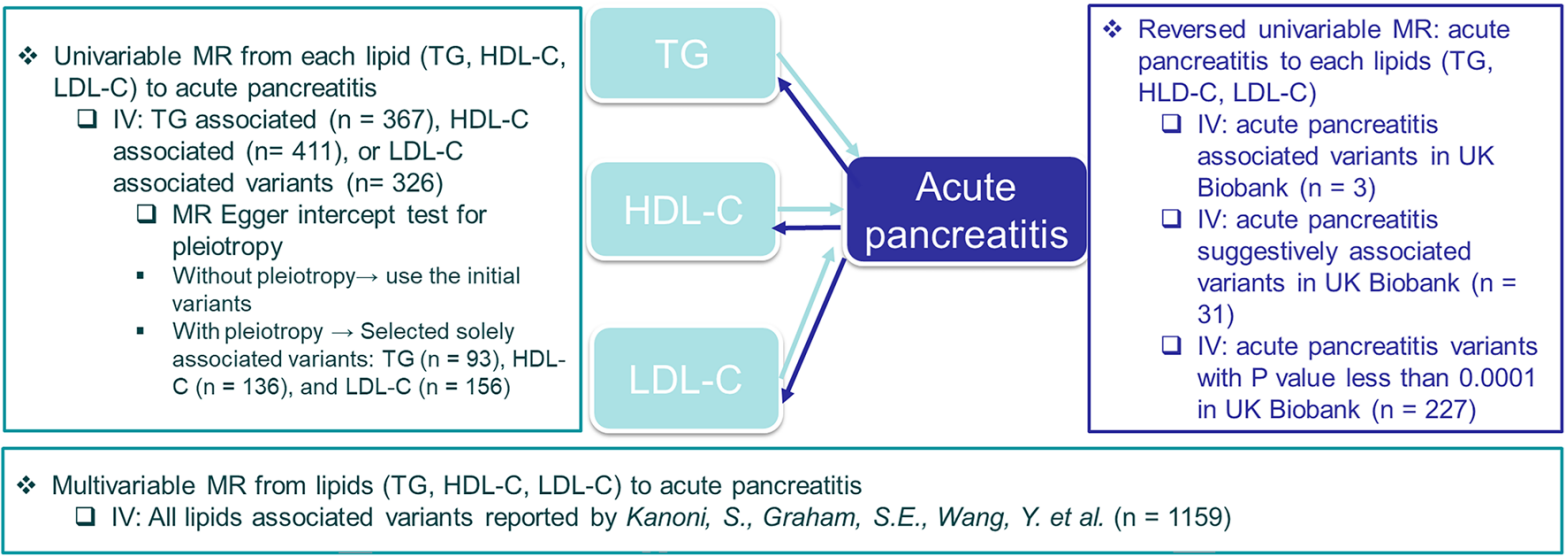
Instrumental variables selections in the bidirectional MR of lipids and acute pancreatitis.

We filtered variants to have a minor allele frequency (MAF) > 1% in the UK Biobank, imputation quality > 0.3, not palindromic (i.e., MAF close to 0.5), and were available in both GLGC and UK Biobank GWAS summary statistics. We obtained multiple distinct variants for pancreatitis from the GWAS in UK Biobank by clumping variants on linkage disequilibrium r^2^ < 0.01 in 1000G Europeans reference panel. We used the independent variants previously reported by GLGC as instrumental variables for lipid levels. We examined the association of genetically predicted lipids on acute pancreatitis in a mutually adjusted model (Supplemental Methods, Fig. 4, Supplemental Table 8). We additionally used the MR Egger intercept test to evaluate potential horizontal pleiotropy^43^ and selected the variants solely associated with each lipid trait (*P*-values < 5e−08 for the target lipid trait, while *P*-values for the other two lipid traits were > 1e−04 [as we assumed there were approximately 500 independent variants associated with one lipid trait, for each of those 500 variants, their associations with other lipids at the Bonferroni corrected significant level should be 0.05/500] in GLGC multi-ancestry results including UK Biobank).

To examine the reverse causation from acute pancreatitis to lipids, we used three sets of variants as instrumental variables: (1) independent variants reached the genome-wide association significant level (*P*-value < 5e−08 and clumping variants on linkage disequilibrium r^2^ < 0.01) in the UK Biobank acute pancreatitis GWAS (Fig. 4 and Supplemental Table 9), (2) independent variants at the suggestive significant level (*P*-values < 1e−05), and (3) independent variants at an even more relaxed significance level (*P*-values < 1e−04, Supplemental Table 9) from the results of UK Biobank (Supplemental Methods).

### Mendelian randomization

We used multivariable two-sample MR to estimate the independent effects of TG, HLD-C, and LDL-C on acute pancreatitis^44^. For univariable MR analysis, we used inverse-variance weighted (IVW) method to aggregate the Wald ratio estimates for each variant-exposure and variant-outcome associations. The Steiger test of directionality was performed to infer whether the assumptions from exposure to outcome was valid^45^. Each genetic instrument F-statistic was calculated to confirm the genetic variants were strongly associated with the exposure (F-statistic > 10). Heterogeneity of summary estimates was estimated using Cochran’s Q test^33^. Horizontal pleiotropy was evaluated by the MR Egger intercept test. We also conducted several sensitivity analyses including Steiger-filtering of plausible reverse causation variants, MR-PRESSO^46^, MR-Egger, Simple mode, weighted median, and weighted mode methods^47^. Moreover, we performed univariable MR for each lipid trait to acute pancreatitis based on the instruments from the *LPL* pathway to validate previous causal relationships between TG and acute pancreatitis. We used R 3.6.0 and TwoSampleMR packages (version 0.5.6) for MR analysis^48^.

### Ethics approval and consent to participate

Analysis on UK Biobank data was performed under application number 42614, covered by the general ethical approval for UK Biobank studies from the National Health Service National Research Ethics. Ethical approval and participant consent were obtained in each of the original studies in the GLGC that generated the summary-level data.

### Supplementary Information

Supplementary Information 1.

Supplementary Table 2.

## Data Availability

Analysis on UK Biobank data was performed under application number 42614. Because of the sensitive individual‐level nature of these data, they are not available to share by the authors but can be accessed by application directly to the UK Biobank. Million Veteran Program (MVP) Summary Results from Non-Sensitive Omics Studies were obtain from dbGaP Accession: phs002453.v1.p1. GLGC summary statistics were obtained from http://csg.sph.umich.edu/willer/public/glgc-lipids2021/.

## Abbreviations

TG: Triglycerides
HDL-C: High density lipoprotein cholesterol
LDL-C: Low density lipoprotein cholesterol
MR: Mendelian randomization
GWAS: Genome wide association studies
bi-MR: Bidirectional Mendelian randomization
GLGC: Global Lipids Genetics Consortium
MVP: Million Veteran Program
MAF: Minor allele frequency
