# Supplementary material for: Lipid levels and risk of acute pancreatitis using bidirectional Mendelian randomization
Source: Sci Rep. 2024 Mar 15;14:6267. doi: 10.1038/s41598-024-56946-x (PMC10942988; doi:10.1038/s41598-024-56946-x)
Supplement: Supplementary file 1 — Supplementary Information 1. [file 41598_2024_56946_MOESM1_ESM.pdf]

Lipids and risk of acute pancreatitis using bidirectional Mendelian Randomization

SUPPLEMENTAL MATERIAL

Contents

Supplemental Methods 2
Supplemental Figure 1. 5
Supplemental Figure 2. 6
References 7

Supplemental Methods

Study Populations

We examined the phenotypic associations between baseline lipids (TG, HDL-C, and LDL-C) and risk of acute pancreatitis in UK Biobank (**Supplemental Figure 2**). The UK Biobank is a large prospective cohort in which 502,406 participants were recruited from 2006 to 2010 with in-depth genetic and health information data[1]. Participants with missing data for TG, HDL-C, or LDL-C, or those receiving chemotherapy or with prevalent chronic kidney diseases were excluded **(Supplemental Figure 2**)[2, 3]. We also excluded 1648 participants with prevalent acute pancreatitis and 421,181 participants remained for the phenotypic association analysis.

Lipid measurements in studies from Global Lipids Genetics Consortium (GLGC) were previously described[4], whereas serum lipids in the UK Biobank were measured by biochemistry blood assays using a standard approach on a Beckman Coulter AU5800[5]. Acute pancreatitis in the UK Biobank diagnosed from hospitalization data and death records by the International Statistical Classification of Diseases and Related Health Problems 10th revisions (ICD-10) codes of K85, K85.0, K85.1, K85.2, K85.3, K85.8, K85.9 or 9th revisions (ICD-9) codes of 5770. The electronic health record (EHR) derived clinical outcomes code (phecode, i.e., all ICD-10 or ICD-10 codes for all Veterans in MVP were extracted and each assigned a phenotype defined by a phecode) 557.1 was used for acute pancreatitis in MVP[6]. Covariates for the phenotypic associations in UK Biobank are defined in **Supplemental Table 7**.

Genetic Data

Genotype data in GLGC was imputed from the Haplotype Reference Consortium or 1000 Genomes Phase 3 that successfully passed variant-level quality control, and details are available elsewhere[4]. The genotype data in the UK Biobank was imputed by the UK10K and 1000 Genomes Phase 3 reference panels for over 480,000 participants who passed the sample-based quality control **(Supplemental Figure 2**) across diverse ancestries[7].

The MVP is a nation-wide cohort launched in 2011 to study the contributions of genetics, lifestyle, and military exposures to health and diseases among US veterans[8]. Blood samples were genotyped using a customized Affymetrix Axiom biobank array (the MVP 1.0 Genotyping Array) and imputed by a hybrid imputation panel comprised of the African Genome Resources panel and 1000G Phase 3 Version 5 panel[9]. Details of the genotyping, quality controls, and imputations are reported elsewhere[10]. There were 11,831 acute pancreatitis cases and 613,206 controls in MVP. These

GWAS results were created as part of the MVP genome-wide PheWAS project[11], a collaboration between the US Department of Veterans Affairs and the US Department of Energy.

Genome-wide association study of pancreatitis

The genetic associations for lipids were obtained from a GWAS meta-analysis of GLGC without the UK Biobank, where TG were natural log transformed and the lipids residuals were inverse normalized. The genetic associations for acute pancreatitis in the UK Biobank were performed using SAIGE[12] v0.45 which accounts for sample relatedness and case-control imbalance by a generalized mixed model adjusted for age, sex, genotyping batch, and the first 10 genetic principal components (PCs). Genetic association analysis of acute pancreatitis in the MVP was also conducted using generalized linear mixed models to account for participant relatedness using a GPU-optimized version of the SAIGE package within each defined ancestry group (non-Hispanic White (EUR), non-Hispanic Black (AFR), Hispanic or Latino (HIS), or Asian (ASN))[13]. Analyses were adjusted for age, sex, and 10 ancestry-specific PCs. Then a multi-ancestry meta-analysis was performed using the inverse-variance weighted method as implemented in GWAMA[14] to generate the acute pancreatitis MVP GWAS results. We used METAL[15] to conduct the fixed-effects meta-analysis of UK Biobank and the MVP.

Selection of instrumental variables

To examine the association of genetically predicted lipids on acute pancreatitis in a mutually adjusted model (e.g., TG and acute pancreatitis association conditional on HDL-C and LDL-C), 1159 variants which were significantly associated with at-least one lipid trait (i.e., MAF> 1% in the UK Biobank, imputation quality>0.3, not palindromic, and P values <5e-08 in GLGC multi-ancestry results including UK Biobank) were selected from the GWAS summary statistics (**Supplemental Table 8**). From those 1159 variants we selected 367 variants for TG (i.e., associated with TG at P values < 5e-08), 411 variants for HDL-C (i.e., associated with HDL-C at P values < 5e-08), and 326 variants for LDL-C (i.e., associated with LDL-C at P values < 5e-08) as individual instruments for each lipid trait, respectively. We additionally selected the variants solely associated with each lipid trait (P-values<5e-08 for the target lipid trait, while P values for the other two lipid traits were > 1e-04 (0.05/500) in GLGC multi-ancestry results including UK Biobank). The variants solely associated with TG, HDL-C, and LDL-C numbered 93, 136, and 156 respectively (**Supplemental Table 8**).

To examine the reverse causation from acute pancreatitis to lipids, three independent variants reached the genome-wide association significant level (P value <5e-08 and clumping variants on linkage disequilibrium r^2<0.01 in 1000G Europeans reference panel) in the UK Biobank acute pancreatitis GWAS and were selected as the instrumental variables (**Supplemental Table 9**). We also selected 31 independent variants at the suggestive significant level (P-values<1e-05)

as instruments in the sensitivity analysis of MR. To include several top signals in the meta-analysis results of UK Biobank and MVP, we selected 227 independent variants at an even more relaxing significance level (P-values < 1e-04, **Supplemental Table 9**) in the UK Biobank as the instruments for the reverse MR sensitivity analysis.

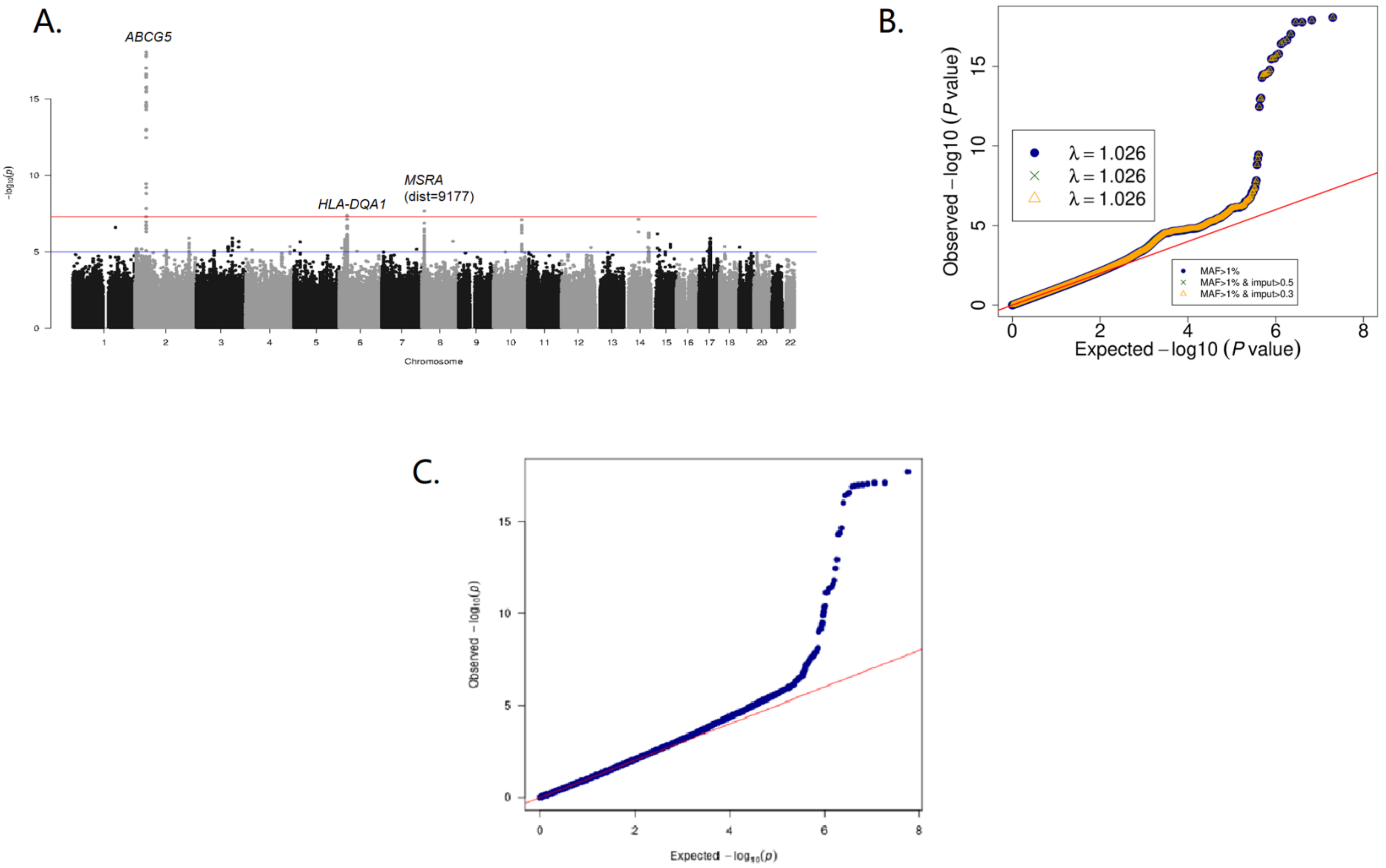

Supplemental Figure 1. Manhattan plot and QQ plot of acute pancreatitis in UK Biobank (panel A, B) and QQ plot for the meta-analysis of UK Biobank and MVP (Panel C)

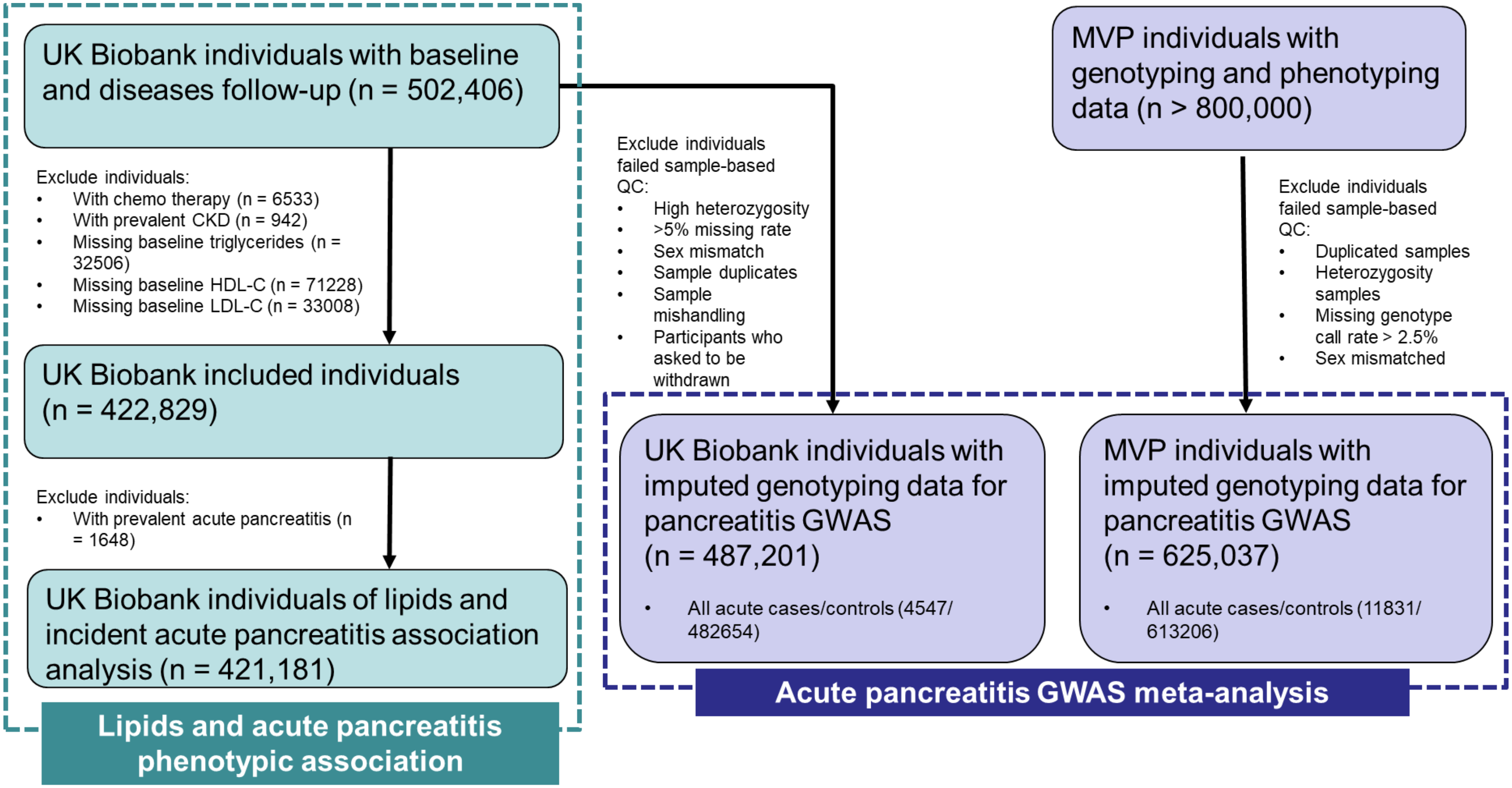

Supplemental Figure 2. Sample sizes of eligible participants in the phenotypic associations of lipids and acute pancreatitis and acute pancreatitis GWAS meta-analysis

References

1. Sudlow C, Gallacher J, Allen N, et al. UK biobank: an open access resource for identifying the causes of a wide range of complex diseases of middle and old age. PLoS Med 2015;12:e1001779.
2. Spagnolo DM, Greer PJ, Ohlsen CS, et al. Acute and Chronic Pancreatitis Disease Prevalence, Classification, and Comorbidities: A Cohort Study of the UK BioBank. Clin Transl Gastroenterol 2022;13:e00455.
3. Whitcomb DC, North American Pancreatitis Study G. Pancreatitis: TIGAR-O Version 2 Risk/Etiology Checklist With Topic Reviews, Updates, and Use Primers. Clin Transl Gastroenterol 2019;10:e00027.
4. Graham SE, Clarke SL, Wu KH, et al. The power of genetic diversity in genome-wide association studies of lipids. Nature 2021;600:675-679.
5. Kanoni S, Graham SE, Wang Y, et al. Implicating genes, pleiotropy, and sexual dimorphism at blood lipid loci through multi-ancestry meta-analysis. Genome Biol 2022;23:268.
6. Aung N, Sanghvi MM, Piechnik SK, et al. The Effect of Blood Lipids on the Left Ventricle: A Mendelian Randomization Study. J Am Coll Cardiol 2020;76:2477-2488.
7. Bycroft C, Freeman C, Petkova D, et al. The UK Biobank resource with deep phenotyping and genomic data. Nature 2018;562:203-209.
8. Gaziano JM, Concato J, Brophy M, et al. Million Veteran Program: A mega-biobank to study genetic influences on health and disease. J Clin Epidemiol 2016;70:214-23.
9. Klarin D, Damrauer SM, Cho K, et al. Genetics of blood lipids among ~300,000 multi-ethnic participants of the Million Veteran Program. Nat Genet 2018;50:1514-1523.
10. Hunter-Zinck H, Shi Y, Li M, et al. Genotyping Array Design and Data Quality Control in the Million Veteran Program. Am J Hum Genet 2020;106:535-548.
11. Verma A, Huffman JE, Rodriguez A, et al. Diversity and Scale: Genetic Architecture of 2,068 Traits in the VA Million Veteran Program. medRxiv 2023:2023.06.28.23291975.
12. Zhou W, Nielsen JB, Fritsche LG, et al. Efficiently controlling for case-control imbalance and sample relatedness in large-scale genetic association studies. Nat Genet 2018;50:1335-1341.
13. Fang H, Hui Q, Lynch J, et al. Harmonizing Genetic Ancestry and Self-identified Race/Ethnicity in Genome-wide Association Studies. Am J Hum Genet 2019;105:763-772.
14. Magi R, Morris AP. GWAMA: software for genome-wide association meta-analysis. BMC Bioinformatics 2010;11:288.
15. Willer CJ, Li Y, Abecasis GR. METAL: fast and efficient meta-analysis of genomewide association scans. Bioinformatics 2010;26:2190-1.
